# Oral lactase for infantile colic: a randomized double-blind placebo-controlled trial

**DOI:** 10.1186/s12887-022-03531-8

**Published:** 2022-08-03

**Authors:** Manish Narang, Dheeraj Shah

**Affiliations:** Division of Pediatric Gastroenterology, Hepatology and Nutrition, Department of Pediatrics, University College of Medical Sciences (University of Delhi) and Guru Teg Bahadur Hospital, Delhi, India

**Keywords:** Crying, Fussy infant, Infantile colic, Lactase, Treatment

## Abstract

**Background:**

Infantile colic is a common problem during the first three months of life. This randomized, double-blind, placebo-controlled trial conducted in an urban hospital in Delhi, India evaluated the efficacy and safety of oral lactase in management of infantile colic.

**Methods:**

One hundred sixty-two clinically healthy infants aged < 5 months age [mean (SD) = 63.5 (30.5) days] fulfilling the Rome-IV diagnostic criteria for infantile colic were enrolled. Eligible children were randomly allocated to receive 5 drops of lactase (600 FCC units/mL) (*n* = 80) or placebo (*n* = 82) mixed with breast milk or formula feed four times a day for a duration of 4 weeks. Primary outcomes were duration of crying or fussing (min/d), and number of days with colic lasting > 3 h/d; secondary outcomes were parental satisfaction and adverse events.

**Results:**

At the end of four weeks, mean (SD) crying or fussing time (min/d) was significantly shorter in infants receiving lactase in comparison to placebo [89.9 (115.2) *vs*.178.5 (153.2); *P* = 0.001]. The mean (SD) number of days with colic was also significantly less in the lactase group as compared to placebo group at the end of the treatment [12.1 (7.8) *vs* 17.6 (8.4); *P* < 0.001]. By the end of 4^th^ week, parental satisfaction in terms of infant’s mood, activity, alertness, comfort and oral intake was better in intervention group. The adverse event profile was comparable between two groups.

**Conclusions:**

Oral lactase treatment in infantile colic results in symptomatic relief in terms of shortening of duration of crying or fussing, and better parental satisfaction.

**Trial registration:**

Clinical trial registry of India (CTRI/2017/12/010930) registered on 20/12/2017.

## Background

Infantile colic – unexplained and inconsolable crying episodes without any identifiable cause in otherwise healthy infants is a common problem [1]. It may result in parental distress, child abuse and early breast-feeding cessation [2]. Currently, there is no well-established treatment of infantile colic. Systematic reviews are available on pharmacological treatment, parent training program, dietary modification and probiotics in prevention on infantile colic [3–8]. Most treatment strategies have failed to document significant improvement [9]. The use of complementary and alternative medicine (CAM) is increasing in various conditions such as cardiovascular disease, diabetes, and infantile colic [10, 11] where there is no definite ‘cure’. CAM strategies in infantile colic include spinal manipulations, herbal medicine and pain-relieving agents, and acupuncture [12]. The evidence for efficacy of pain-relieving agents like simethicone, dicyclomine, cimetropium bromide, and herbal medicines for treatment of infantile colic is sparse [3]. The effectiveness and safety of parent training programme for managing infantile colic is also inconclusive [5]. There is no clear evidence that probiotics such as *Lactobacillus*, *Bifidobacterium* and *Streptococcus* are more effective than placebo at preventing infantile colic; however, daily crying time appeared to reduce with probiotic use compared to placebo in babies with infantile colic [7].

Infantile colic has been postulated to be caused due to painful intestinal contractions, lactose intolerance, altered gut microbiota, aerophagy, food hypersensitivity and behavioral factors [13]. Presence of low-grade systemic inflammation as evident by increased systemic and gut inflammatory biomarkers along with abnormal gut microbial composition has also been documented to be associated with infantile colic [14, 15].

Transient lactose intolerance is considered an immediate cause of infantile colic. Research is still ongoing to investigate the role of lactose in infantile colic [16, 17]. Lactase is located on the brush border of the small intestine which hydrolyzes lactose to glucose and galactose. Undigested lactose produces lactic acid and hydrogen in infants, which may cause bloating, diarrhea and flatulence [9]. As the only diet for infants below 6 months is milk, lactase enzyme supplementation with milk may have the propensity to mitigate these colic symptoms. Two randomized, double-blind, placebo-controlled crossover studies showed significant reduction in crying time through incubation of milk feed (breast or bottle) with lactase, an enzyme that digests the disaccharide lactose [16, 17]. However, both these trials did not utilize currently used stringent definition of colic as per Rome criteria [18] and had a small sample size. Lack of a definite theory regarding the cause of infantile colic and very scarce research on the role of lactase in treating infantile colic call for further research assessing the efficacy of lactase enzyme in infantile colic treatment. Based on the knowledge about the role of lactose intolerance in infantile colic due to an immature digestive system, we aimed to evaluate the efficacy and safety of oral lactase enzyme supplements in management of infantile colic with primary objectives of comparing crying or fussing duration and number of colic days, and secondary objectives of comparing the parent satisfaction and adverse effects in children receiving lactase or placebo.

## Methods

This randomized, double-blind, placebo-controlled trial was conducted from February 2018 to February 2020 in the division of Pediatric Gastroenterology, Hepatology and Nutrition, Department of Pediatrics at a medical school affiliated hospital in Delhi, India. The study protocol was approved by the Institutional Ethics Committee – Human Research (IEC-HR) of the institute, and written informed consent was obtained from the parent(s) or caregiver of included infants.

### Study population

Infants (age < 5 mo) fulfilling the Rome-IV diagnostic criteria for infantile colic [18] with no evidence of acute/chronic illness were eligible for inclusion. Rome-IV diagnostic criteria defines infantile colic as recurrent and prolonged periods of crying, fussing or irritability reported by caregivers that occur without any obvious cause in an infant who is less than 5 months of age when symptoms start and stop and cannot be prevented or resolved by caregivers; and no evidence of failure to thrive, fever or illness”. For clinical research purpose, diagnosis of infantile colic also includes both of the following “(i) caregiver reports infant has cried or fussed for 3 or more hours per day during 3 or more days in 7 days in a telephone or face-to-face screening interview; and (ii) total 24-h crying plus fussing in the selected group of infants is confirmed to be 3 h or more when measured by at least one prospectively kept, 24-h behaviour diary”. Parents who reported their infant to be crying excessively were screened on outpatient basis. Colic was then defined by study pediatricians in those infants whose cry/fuss behaviors, as recorded in the diary for preceding 7 days, fulfilled Rome IV criteria. Infants born before completing 34 weeks of gestation or birth weight < 2500 g, and inadequate weight gain (< 125 g/wk) were excluded from the trial. Infants with known gastrointestinal disorders, previous abdominal surgery, known galactosemia or diabetes mellitus were also excluded from the study.

### Clinical assessment

Medical evaluation of enrolled infants was performed by pediatricians, which included detailed medical, family and social history, complete physical and systemic examination, and review of past records. Anthropometric parameters such as weight, length and head circumference were recorded. Blood investigations at enrolment included complete blood count, blood sugar, serum bilirubin, alanine- and aspartate-aminotransferase, urea and electrolytes. An ultrasonography of the abdomen was performed for infants at the clinical discretion of study investigators.

### Randomization and blinding

Sequence based on block randomization with variable block size was generated using an online calculator (available at www.randomization.com). This sequence was concealed in sequentially numbered opaque sealed envelopes in form of multiple codes (3 each for drug and placebo) to avoid guessing of code by investigators. Each bottle (15 mL) containing lactase or placebo was coded with one of these six codes. Randomization sequence and codes were generated by a person not directly involved with the study. The bottles were coded by the pharmacy. Parents of participants, study investigators and outcome assessors were blinded to the treatment assigned. The intervention and placebo were packaged identically, and were designed to be similar in taste (sweet); both were odorless and colorless. At the time of statistical analysis, three codes for drug or placebo were combined, and analysis was carried out between two intervention groups. The final code was broken at the end of the statistical analysis and tabulation of the results.

### Intervention and comparison

Enrolled infants were randomly assigned to receive five drops (0.2 mL) of the lactase enzyme preparation (Yamoo drops, Walter Bushnell Pvt. Ltd; 600 FCC units/mL) or placebo (clear, colourless, odourless syrupy liquid with the same ingredients as lactase enzyme preparation except for lactase). In breastfed babies, mothers were instructed to express the 10 mL fore-milk, which has the highest lactose content [17], into a tea-spoon or other sterile container, and then five drops of the lactase or placebo were added. After 30 min this mixture was given to the baby and then breast-feeding continued as usual. In formula-fed babies, mothers were instructed to add five drops of lactase or placebo to 50 mL of infant formula (when it was warm). She was asked to wait for 30 min, shake the formula occasionally and then feed the baby. Both formulations were administered in four times in a day for 28 days. During the study, parents were instructed to refrigerate the drug when it was not in use. Parents were instructed to continue their usual feeding habits and asked to avoid any additional medication apart from the formulation supplied to them. They were advised to use cuddling, rocking, and other comforting methods to pacify the infants if needed.

### Monitoring and follow-up

Parents were instructed to complete 24-h diaries of infant crying and other behavior based on the criteria laid by Barr et al*.* for 28 consecutive days following enrolment [19]. The crying duration was recorded to an accuracy of 5 min using the ‘Time rulers’ in the diary card as recommended by Barr et al. Parents were also instructed to record stool characteristics and frequency, and any solicited (vomiting/milk regurgitation, diarrhea and constipation) or unsolicited adverse events (AEs) observed on each day of the study. Parental satisfaction based on their perception of child’s mood, activity, alertness, oral intake, vomiting and comfort was assessed on a five-point Likert scale. The responses on Likert scale were 1: getting worse, 2: no improvement, 3: some improvement, 4: marked improvement, and 5: completely well [20]. Enrolled infants were called for follow-up visits on Day 7, 14, 21 and 28. Symptom diary and infant’s weight were checked at every visit. All the bottles were collected from the parents at the end of the study. Adherence was assessed by examining the volume of medication left; residual volume being more than 20% of the calculated volume (dose dispensed – dose prescribed) was considered non-compliance.

### Study outcomes

The primary outcomes of the study were: duration of crying or fussing (that starts and stop without obvious cause) in minutes/days during 4 weeks of use of drug/placebo and number of days with colic (that lasts > 3 h/day) [21]. The secondary outcomes included parental satisfaction (on a Likert scale) and occurrence of AEs (*e.g.* milk regurgitation, vomiting, diarrhea, constipation). AEs such as illness signs, or symptoms that occurred or got worse during the course of the study were assessed through parental interview after examination of their daily records maintained in the dairy.

### Sample size and statistical analysis

Sample size was calculated based on data from a previous study [22] evaluating efficacy of a probiotic in 167 infants (age < 3 months) with colic, which documented that mean (SD) duration of crying or fussiness was 191 (103) min/day in placebo group. To detect a reduction of 30% in duration of crying and fussing at the end of 4 weeks by the use of lactase drops, the sample size was calculated to be 73 infants in each group with type I error as 5% and power of study as 90%. Accounting for 10% attrition, total of 160 (80 in each group) infants were planned to be enrolled.

Data related to daily crying and fussing duration were presented for every week separately. For overall duration of crying during study period, data for the whole 4-week follow-up period was combined and averaged. Descriptive statistics were used to describe continuous variables as mean (SD). The crying and fussing time was compared with Student t test, whereas parental satisfaction was compared in two groups by Mann Whitney test for every week as well as for the entire duration. Proportions were compared using Chi square test or Fischer Exact test as applicable. A *P* value of less than 0.05 was considered to be statistically significant.

## Results

**Fig. 1:**
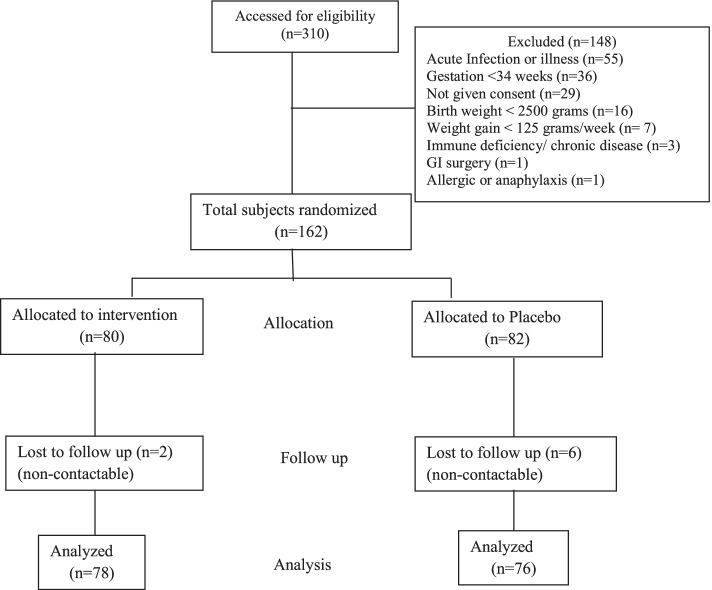
Study flow chart

A total of 162 children (99 boys and 63 girls) were included in the study; 154 completed the follow-up of 4 weeks (Fig. 1). Baseline characteristics of the infants enrolled in the study in Table 1 show crying duration in study and control group was comparable (Table 1). Over three-fourths of infants in both groups were exclusively breast fed. All infants not on breast feeds were receiving normal term infant formula feeds.

**Table 1:** Baseline characteristics of the infants enrolled in the study

Variable	Intervention (*n* = 80)	Placebo (*n* = 82)	*P*-value
Age (mo), mean (SD) ≤ 3 mo, n (%)	2.08 (0.99) 66 (82%)	2.16 (1.04) 65 (81%)	0.612 0.601
Male gender, n (%)	48 (60%)	51 (62%)	0.774
Exclusively breastfed n (%)	60 (75%)	66 (80%)	0.401
Birth weight (g) Mean (SD)	2986.9 (403.4)	2909.8 (365.6)	0.204
Enrolment weight (g) Mean (SD)	4509.9 (844.9)	4665.4 (1081.15)	0.309
Length (cm) Mean (SD)	56.2 (3.7)	56.2 (3.7)	0.899
Hemoglobin (g/dL) Mean (SD)	12.9 (1.7)	13.1 (1.9)	0.616
Crying or fussing duration (min/d); Mean (SD)	378.4 (88.9)	373.05 (100.34)	0.721
Crying duration (min/d); Mean (SD)	206.63 (47.8)	211.0 (59.1)	0.607
Fussing duration (min/d); Mean (SD)	171.9 (62.4)	165.2 (57.0)	0.481

Table 2 presents the results of crying and fussiness at the end of 4 weeks and also after the end of each week. The mean daily duration of crying or fussing showed a progressive decline from first to fourth week in both the groups but was significantly lesser in infants receiving lactase in comparison to those receiving placebo, at the end of every week of assessment. Considering crying and fussing duration for all 4 wks of study, the mean (SD) duration of crying and fussing was 155.1 (101.4) min while in case of placebo group it was 234.1 (127.1) min. Infants receiving lactase had a mean reduction in crying or fussing time by 35.1–52.2 min/day. The reduction in crying or fussing duration was higher during 3^rd^ and 4^th^ week of intervention (Table 2). After a period of four weeks, better parental satisfaction in terms of perception of their child’ mood, alertness, activity and oral intake was observed in infants receiving lactase in comparison to placebo (Table 3).

**Table 2:** Evaluation of primary outcome variables during the treatment in both the groups

Outcome	Lactase Mean (SD)	Placebo Mean (SD)	Mean Difference (95% CI)	*P value*
**Week 1**				
Crying or fussing duration (min/d)	240.7 (109.4)	301.7 (112.0)	-61.1 (-95.3, -26.6)	0.001
Crying duration (min/d)	137 (59.3)	172.2 (66.4)	-35.1 (-54.7, -15.6)	0.001
Fussing duration (min/d)	103.7 (58.5)	130.9 (56.3)	-27.1(-44.9, -9.3)	0.003
**Week 2**				
Crying and fussing duration (min/d)	174.8 (120.3)	241.7 (126.4)	-66.8 (-105.2, -28.4)	0.001
Crying duration (min/d)	98.6 (67.1)	138.8 (76.5)	-40.2 (-62.6, -17.7)	0.001
Fussing duration (min/d)	79.2 (59.7)	105.8 (59.5)	-26.6 (-45.1, -8)	0.005
**Week 3**				
Crying and Fussing duration (min/d)	110.3 (114.7)	199.4 (144.8)	-89.1 (-130.4, -47.7)	0.001
Crying duration (min/d)	61.3 (61.8)	113.5 (82.9)	-52.2 (-75.4, -29)	0.001
Fussing duration (min/d)	49.0 (57.5)	86.0 (67.1)	-36.9 (-56.7, -17.2)	0.001
**Week 4**				
Crying and fussing duration (min/d)	89.9 (115.2)	178.5 (153.2)	-88.6 (-131.8, -45.4)	0.001
Crying duration (min/d)	49.9 (63.54)	100 (86.0)	-50 (-74.1, -25.9)	0.001
Fussing duration (min/d)	39.9 (55.2)	78.5 (71.1)	-38.5 (-58.9, -18.2)	0.001
**Average (over 4 weeks)**				
Crying or fussing duration (min/d)	155.1 (101.5)	234.1 (127.1)	-79.1 (-114.7, -43.4)	< 0.001
Crying duration (min/d)	86.55 (54.8)	132.4 (72.8)	-45.9 (-66.0, -25.9)	< 0.001
Fussing duration (min/d)	68.6 (51.3)	101.8 (59.2)	-33.2 (-50.4, -16.0)	< 0.001
Colic days	12.1 (7.8)	17.6 (8.4)	-5.5 (-8.0, -2.3)	< 0.001

**Table 3:** Parental satisfaction score and adverse events during four weeks of treatment in both the groups

Outcome	Lactase Median (IQR) (*n* = *78*)	Placebo Median (IQR) (*n* = *76*)	*P value*
**At the end of Treatment**			
Mood	5 (4, 5)	3 (2, 4)	< 0.001
Activity	5 (4, 5)	4 (3, 5)	0.005
Alertness	5 (4, 5)	4 (3, 5)	0.002
Comfort	5 (4, 5)	3 (2, 4)	< 0.001
Vomiting	5 (4, 5)	4 (3, 5)	0.021
Fluid Intake	5 (4, 5)	4 (3, 5)	0.033
**Adverse events**	12	31	0.27
Constipation	0	1	
Diarrhea	3	3	
Vomiting	4	9	
Regurgitation of feeds	3	11	
Eating poorly than usual	1	0	
Cutaneous reaction	0	1	
Fever	1	4	
Others	0	2	

The residual volume of drug was within 20% of that expected on basis of calculated dose in all the participants. Overall, the treatment was well tolerated in both the groups; 43 infants had mild AEs such as vomiting, regurgitation of feeds, constipation and diarrhea that resolved (Table 3) without the need of any other medication or hospitalization.

## Discussion

In this randomized controlled trial, we documented a significant reduction in crying and fussing duration in children receiving lactase drops added to milk formula or breast milk prior to feeding in comparison to those receiving placebo during the entire 4-week treatment period. This also translated into a lesser number of days with colic, and better parental satisfaction in terms of positive change in child’ mood, alertness, activity and oral intake. The treatment with lactase was well tolerated by all the infants as no serious adverse event was reported during the treatment.

Regarding the treatment of infantile colic, earlier studies reported decreased crying duration when lactase preparation was administered as an intervention [16, 17, 23]. A randomized, double-blind, cross-over trial of lactase and placebo drops added to milk formula of 13 infants with infantile colic showed significant reduction in crying time [16]. A double-blind randomized placebo-controlled crossover study in 53 infants with colic resulted in significant reduction in both cry time and breath hydrogen with pre-incubation of feed with lactase [17]. A double-blind placebo controlled randomized clinical trial conducted in 104 infants aged 0–6 months with infantile colic also showed significant improvement in duration of crying in infants who received lactase enzyme supplement before each breast-feed [23]. However, lactase had no significant effect on crying duration when given orally after feeds [24]. It was assumed that lactase gets inactivated in the stomach when given after feed. In contrast, lactase showed improvement in other children when given with food [25]. Our results suggested that lactase drops when added to milk formula or breastfeed prior to feeding resulted in a significant reduction in crying in infant with colic. This suggests the lactase drops may require prior incubation with milk and helps in reduction of colic days.

Theories proposed relating various infantile colic factors can be classified as gastrointestinal or non-gastrointestinal. The gastrointestinal causes include lactose intolerance, altered gut micro-organisms, increased motilin receptors or cow milk hypersensivity. The non-gastrointestinal causes include behaviour causes or altered child-parent interaction. As majority of infants in our study showed reduction of crying duration after administration of lactase drops, lactose intolerance may have significant contribution to causation of infantile colic. The risk of symptoms after lactose ingestion depends on the dose of lactose, lactase expression, intestinal flora, and sensitivity of gastrointestinal tract [26]. The capacity of colonic microbes to process lactose can adapt to increased flux of lactose into the colonic lumen [27]. Colonic adaptation occurs mainly in lactase-deficient individuals and is possibly responsible for the increased tolerance to lactose after a lactose-feeding period. Spontaneous recovery of colic by 5–6 months in infants who continue on milk diet alone also may be related to adaptation of colonic microbes to continuing lactose stimulation of gut. However, there might be sub-group of lactose deficient infants where colonic adaptation does not occur to a desired extent and their lactase level further depletes. This might be the reason for higher quantum of benefit in 3^rd^ and 4^th^ week of supplementation in our study. However, we do not have direct evidence to substantiate this hypothesis.

Infantile colic can also be viewed as a behavioral problem. It may result into inadequate parental reactions, or parental distress or depression. This may also affect the parent–child relationship [28]. Better parental satisfaction in those receiving lactase in our study might help to address these problems, but there are no similar data to compare our results with. Our study showed a positive change in parental satisfaction for management of infantile colic. On Day 28, an improvement was reported in child’s mood, activity and alertness. Their feed intake also increased in both the groups. We observed no serious AEs in both the groups, regurgitation of feeds and diarrhea were the most common AEs with mild effects. A prior study also demonstrated the safety of lactase for the management of infantile colic without any AEs [29].

Our study generated some important findings in real world scenario and had strengths like use of stringent criteria to define colic, placebo-controlled design, focus on functional outcomes, and maintenance of symptom dairy for recording by parents throughout the study thus minimizing recall bias. Parents/caregivers who provided the intervention and assessed the outcome were blinded. The limitation of our study includes a short period of follow-up of 4 weeks that precluded us to comment on any persistent effect of intervention beyond 4 weeks. Moreover, we did not perform hydrogen breath test to investigate for lactose intolerance in study infants, and also did not measure it during follow-up. This precluded analysis of any association between relief of colic symptoms in these neonates and presence of demonstrable lactose intolerance by breath tests. The application of lactase enzyme supplement would improve infant colic due to lactose intolerance, but the supplement will have no impact on infantile colic caused by reasons other than lactose intolerance. As infantile colic is a complex multifactorial problem due to lactose intolerance, altered gut microbiota, aerophagy, food hypersensitivity and behavioral factors, and a large majority of included infants were breast-fed, the results of this study may only be applicable to the subgroup of predominantly breast-fed infants where lactose intolerance is a major causative factor. This study has reported the symptomatic relief in infant crying by lactase supplement for shorter period of time. However, long-term follow-up study is a must to determine the efficacy of lactase supplements correctly [6].

Regarding direct comparison of lactase supplement with other interventions for colic, a previous published study [29] suggested that APT198K (xyloglucan plus heat-killed *Lactobacillus reuteri* SGL01 and *Bifidobacterium brevis* SGB01) was more efficacious than lactase supplement. This was randomized, multicenter, open-label, parallel group, active-controlled study, in 46 infants aged 3–16 weeks with infantile colic, receiving APT198K or a lactase dietary supplement for 10 days. Number and duration of crying episodes decreased significantly versus baseline in both groups. The mean duration of crying per episode was significantly shorter in the APT198K group compared with the lactase group from day 8 to day 11. We did not compare lactose against any other intervention, and thus cannot comment on its superiority or otherwise over other existing interventions such as probiotics.

As all included infants in this study were born at term with adequate birth weight, most were exclusively breast fed and were thriving well, these results may not be generalizable to preterm, low birth weight babies and those receiving formula/bottle feeds. It is possible that in bottle- or formula-fed infants, aerophagy rather than lactase intolerance is more likely cause of colic, and lactase may not work as well. Also, as Rome IV criteria for diagnosis of colic uses stringent parameters, these results should not be extrapolated to any infant with reported excessive crying, unless these colic-defining criteria are satisfied. This may form the basis for further investigation and different treatment options. Further, large scale trials in different sub-groups of infants are needed to support the routine use of lactase supplement in infantile colic. Moreover, as the process of adding lactase and waiting for 30 min may be inconvenient for both mother and baby, alternative modes of administration such as intermittent direct administration of oral lactase (without adding to breast feeds) or waiting for shorter duration after adding lactase to the milk needs to be explored.

We conclude that oral lactase drops may result in significant symptomatic relief in infantile colic in terms of reducing the crying or fussing duration and number of colic days, resulting in better parental satisfaction. A short course of oral lactase drops may be offered to infants with colic who fit the Rome IV diagnostic criteria.

## Data Availability

The datasets used and/or analysed during the current study are available from the corresponding author on reasonable request.

## References

[1] Savino F (2007). Focus on infantile colic. Acta Paediatr.

[2] Howard CR, Lanphear N, Lanphear BP, Eberly S, Lawrence RA (2006). Parental responses to infant crying and colic: the effect on breastfeeding duration. Breastfeed Med.

[3] Biagioli E, Tarasco V, Lingua C, Moja L, Savino F (2006). Pain-relieving agents for infantile colic. Cochrane Database Syst Rev..

[4] Dobson D, Lucassen PLBJ, Miller JJ, Vlieger AM, Prescott P, Lewith G (2012). Manipulative therapies for infantile colic. Cochrane Database Syst Rev..

[5] Gordon M, Gohil J, Banks SSC (2019). Parent training programmes for managing infantile colic. Cochrane Database Syst Rev..

[6] Gordon M, Biagioli E, Sorrenti M, Lingua C, Moja L, Banks SSC, Ceratto S, Savino F (2018). Dietary modifications for infantile colic (Review). Cochrane Database Syst Rev..

[7] Ong TG, Gordan M, Banks SSC, Thomas MR, Akobeng AK (2019). Probiotics to prevent infantile colic. Cochrane Database Syst Rev..

[8] Roberts DM, Ostapchuk M (2004). O'brien JG. Infantile colic Am Fam Physician.

[9] Cohen-Silver J, Ratnapalan S (2009). Management of infantile colic: a review. Clin Pediatr.

[10] Nayebi N, Esteghamati A, Meysamie A, Khalili N, Kalinejad N, Emtiazy M, Hashempur MH (2019). The effects of a Melissa officinalis L. based product on metabolic parameters in patients with type 2 diabetes mellitus: A randomized double-blinded controlled clinical trial. J Complement Integr Med..

[11] Mahdavi-Roshan M, Salari A, Emaminejad S, Parvinroo S. The effect of Oxymel syrup on some cardiovascular risk factors in overweight and obese people: A randomized controlled trial study. Trad Integr Med. 2021;6(3):204–15.

[12] Perry R, Leach V, Penfold C, Davies P (2019). An overview of systematic reviews of complementary and alternative therapies for infantile colic. Syst Rev.

[13] Sarasu JM, Narang M, Shah D (2018). Infantile colic: an update. Indian Pediatr.

[14] Partty A, Kalliomaky M, Salminen S, Isolauri E (2017). Infantile colic is associated with low-grade systemic inflammation. J Pediatr Gastroenterol Nutr.

[15] Sung V (2018). Infantile colic. Aust Prescr.

[16] Kearney PJ, Malone A, Hayes T, Cole M, Hyland M (1998). A trial of lactase in the management of infant colic. J Hum Nutr Diet.

[17] Kanabar D, Randhawa M, Clayton P (2001). Improvement of symptoms in infant colic following reduction of lactose load with lactase. J Hum Nutr Diet.

[18] Zeevenhooven J, Koppen IJ, Benninga MA (2017). The new Rome IV criteria for functional gastrointestinal disorders in infants and toddlers. Pediatr Gastroenterol Hepatol Nutr.

[19] Barr R, Kramer M, Boisjoly C, Mcvey-White L, Pless I (1988). Parental diary of infant cry and fuss behaviour. Arch Dis Child.

[20] Joshi A, Kale S, Chandel S, Pal DK (2015). Likert scale: explored and explained. Br J Applied Sci Technol.

[21] Mai T, Fatheree NY, Gleason W, Liu Y, Rhoads JM (2018). Gastroenterol Clin North Am.

[22] Sung V, Hiscock H, Tang ML, Mensah FK, Nation ML (2014). Treating infant colic with the probiotic Lactobacillus reuteri: double blind, placebo controlled randomised trial. BMJ.

[23] Ahmed M, Billoo AG, Iqbal K, Memon A (2018). Clinical efficacy of lactase enzyme supplement in infant colic: a randomised controlled trial. J Pak Med Assoc.

[24] Swagerty DL, Walling AD, Klein RM (2002). Lactose intolerance. Am Fam Physician.

[25] Treem WR (1994). Infant colic. a pediatric gastroenterologist's perspective. Pediatr Clin North Am..

[26] Misselwitz B, Pohl D, Fruhauf H, Fried M, Vavricka SR, Fox M (2013). Lactose malabsorption and intolerance: pathogenesis, diagnosis and treatment. United European Gastroenterol J..

[27] Forsgård RA (2019). Lactose digestion in humans: intestinal lactase appears to be constitutive whereas the colonic microbiome is adaptable. Am J Clin Nutr.

[28] Akman I, Kuşçu K, Özdemir N, Yurdakul Z, Solakoglu MI (2006). Mothers’ postpartum psychological adjustment and infantile colic. Arch Dis Child.

[29] Vandenplas Y, Bacarea A, Marusteri M, Bacarea V, Constantin M, Manolache M (2017). Efficacy and safety of APT198K for the treatment of infantile colic: a pilot study. J Comp Eff Res.

